# Robot-Assisted Spleen-Preserving Distal Pancreatectomy for an Epithelial Cyst Arising from an Intrapancreatic Accessory Spleen: A Case Report

**DOI:** 10.70352/scrj.cr.26-0171

**Published:** 2026-05-13

**Authors:** Masaki Ueno, Fumitoshi Hirokawa, Yoshinobu Shigekawa, Masamichi Kimura, Hirotaka Tabata, Masaaki Deguchi, Hidemichi Kuroiwa, Yoshihiko Hoshida, Mikihito Nakamori

**Affiliations:** 1 Department of Gastrointestinal Surgery, National Hospital Organization Osaka Minami Medical Center, Kawachinagano, Osaka, Japan; 2 Department of Pathology, National Hospital Organization Osaka Minami Medical Center, Kawachinagano, Osaka, Japan

**Keywords:** intrapancreatic accessory spleen, epithelial cyst, spleen-preserving distal pancreatectomy, robot-assisted surgery

## Abstract

**INTRODUCTION:**

Epithelial cysts arising from an intrapancreatic accessory spleen (ECIPAS) are rare and often misdiagnosed as pancreatic cystic neoplasms because their imaging features frequently overlap. When malignancy cannot be ruled out, surgical resection is generally indicated, and minimally invasive approaches have become an appropriate treatment option. We report a case of ECIPAS initially suspected to be a mucinous cystic neoplasm (MCN), successfully treated with spleen-preserving distal pancreatectomy using the hinotori Surgical Robot System.

**CASE PRESENTATION:**

A 63-year-old woman was incidentally found to have an 18-mm cystic lesion in the pancreatic tail during preoperative imaging for laparoscopic cholecystectomy 2 years earlier. The lesion gradually enlarged to 30 mm over 2 years of serial follow-up. MRI demonstrated a “cyst-in-cyst” morphology without communication with the main pancreatic duct. Contrast-enhanced CT revealed a thick-walled cyst with internal septal calcifications but no solid components. Serum carbohydrate antigen 19-9 and carcinoembryonic antigen levels were within normal limits. Based on the imaging features and lesion growth, we suspected MCN and indicated surgical resection. Consequently, robot-assisted spleen-preserving distal pancreatectomy (Robo-SPDP) was performed using the hinotori system, following the Kimura technique. The procedure was completed safely without any intraoperative complications. Histopathological examination revealed a cyst lined by flattened to columnar epithelium, with an underlying splenic sinus structure confirmed by CD8 immunostaining. Ovarian-type stroma was absent, excluding MCN and establishing a diagnosis of ECIPAS. The postoperative course was uneventful, and the patient was discharged on POD 10.

**CONCLUSIONS:**

This case highlights the importance of considering ECIPAS in the differential diagnosis of pancreatic cystic lesions, particularly when imaging findings resemble cystic neoplasms. When malignancy cannot be ruled out, surgical resection is recommended and minimally invasive spleen-preserving distal pancreatectomy is a reasonable therapeutic option. Furthermore, robotic assistance appeared to be a safe and effective surgical approach facilitating precise dissection and reliable preservation of the splenic vessels.

## Abbreviations

CA19-9
: carbohydrate antigen 19-9
CEA
: carcinoembryonic antigen
CECT
: contrast-enhanced CT
ECIPAS
: epithelial cysts arising from an intrapancreatic accessory spleen
IPAS
: intrapancreatic accessory spleens
MCN
: mucinous cystic neoplasm
Robo-SPDP
: robot-assisted spleen-preserving distal pancreatectomy
SPA
: splenic artery
SPV
: splenic vein
SPIO
: super paramagnetic iron oxide

## INTRODUCTION

An accessory spleen is a relatively common anatomical variant, observed in approximately 10% of patients at autopsy.^1,2)^ Among these, 80% are located in the splenic hilum, whereas 17% arise in the pancreatic tail.^2,3)^ Although typically asymptomatic and incidentally detected, an IPAS may rarely have cystic changes. In such a case, it is sometimes difficult to differentiate it from cystic tumors of pancreatic origin.^4)^ Given that cystic tumors of pancreatic origin have a wide spectrum of entities, ranging from benign to malignant diseases, careful diagnostic evaluation is required.

Herein, we report a rare case of an ECIPAS, which was initially misdiagnosed preoperatively as an MCN due to its gradual increase in size and radiologic characteristics. In this report, we address the challenge of accurate preoperative diagnosis of ECIPAS. Furthermore, we successfully managed this diagnostically complex cystic tumor using minimally invasive surgery: robot-assisted spleen-preserving distal pancreatectomy (Robo-SPDP) performed with the hinotori Surgical Robot System (Medicaroid, Hyogo, Japan) following the Kimura technique. This system is domestically developed in Japan and is characterized by a docking-free design that enables versatile port utilization for the patient-side surgeon during robotic procedures. We highlight its unique features and potential for improving surgical outcomes in such diagnostically complex cystic tumors.

## CASE PRESENTATION

**Fig. 1:**
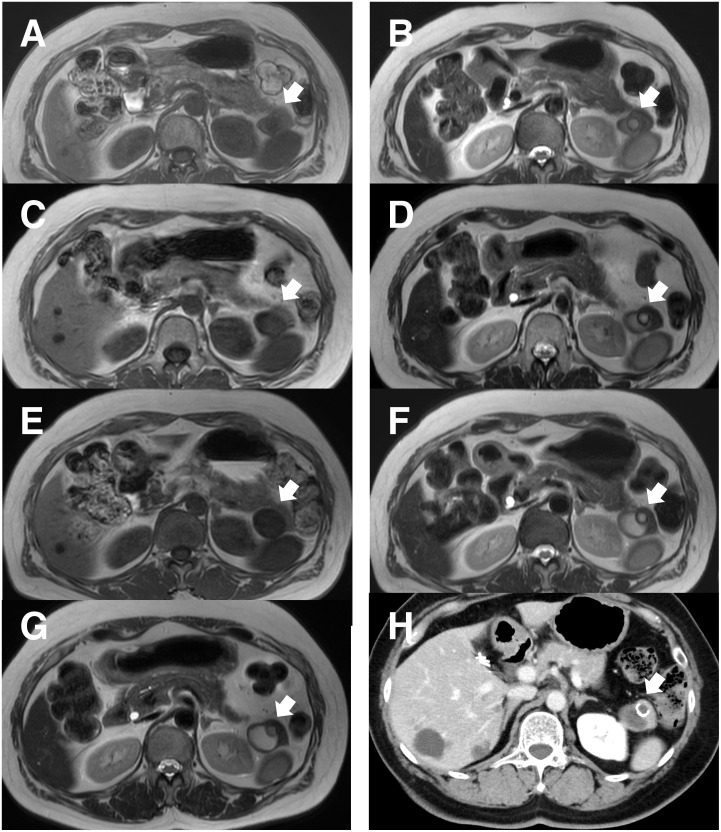
Serial image findings. (**A**, **B**) Initially, the tumor was a 13-mm cystic tumor with hypointense T1 (left panel) and slightly hyperintense T2 signals (right panel), displaying a “cyst-in-cyst” appearance (arrow). (**C–F**) Annually, the size gradually increased in both T1 (left panel) and T2 (right panel) images (arrow). (**G**) Finally, the size became 30 mm in diameter on T2 image, while the morphology remained unchanged (arrow). (**H**) CECT image confirmed a thick capsule with septal calcifications (arrow) but no solid components. CECT, contrast-enhanced CT

A 63-year-old woman was incidentally found to have an asymptomatic 18-mm cystic lesion in the pancreatic tail during preoperative imaging for laparoscopic cholecystectomy performed for cholelithiasis 2 years earlier (**Fig. 1A** and **1B**). MRI demonstrated that the cystic tumor originally appeared as a hypointense lesion on T1-weighted images and slightly hyperintense on T2-weighted images, exhibiting a characteristic “cyst-in-cyst” appearance. Postoperatively, serial imaging follow-up revealed gradual enlargement of the cyst while maintaining its morphology (**Fig. 1C**–**1F**). During MRI follow-up, no communication with the main pancreatic duct was observed. Two years later, MRI showed that the lesion had increased to 30 mm, raising suspicion of a potentially malignant cystic neoplasm (**Fig. 1G**). CECT confirmed a 30-mm cystic lesion with a thick capsule and internal septal calcifications, but no solid components or lymphadenopathy (**Fig. 1H**).

On admission, her height was 154 cm and BMI was 25.3 kg/m^2^. Physical examination revealed a soft, non-tender abdomen with no palpable masses. Laboratory tests demonstrated normal liver and pancreatic enzyme levels. Serum CEA and CA19-9 were within normal limits and their values were 0.7 ng/mL and 13.7 U/mL, respectively.

Based on the imaging features, patient demographics, and interval lesion growth, MCN was suspected. Given the potential for malignancy, surgical resection was recommended. In the absence of invasive features, a minimally invasive surgery was considered reasonable, and Robo-SPDP was selected as an appropriate diagnostic and therapeutic option.

**Fig. 2:**
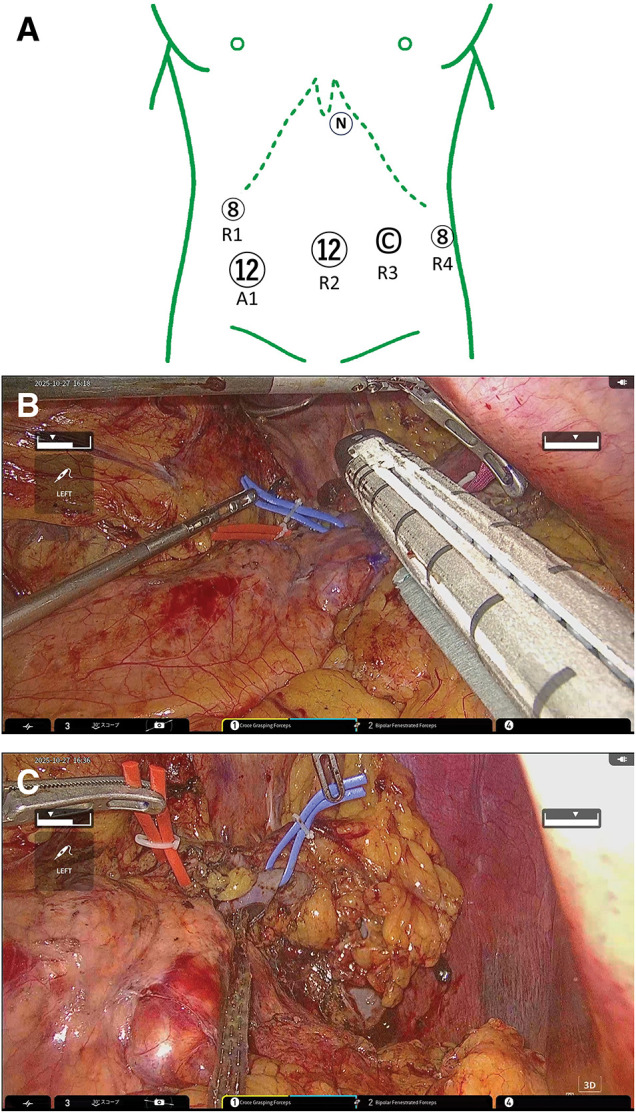
Operative findings. (**A**) A total of 5 trocars were inserted, including one for the patient-side surgeon. (**B**) During pancreatic parenchyma stapling, the R4 was temporarily removed to allow insertion of the stapler by a patient-side surgeon. (**C**) The SPDP was completed using the Kimura technique. A, assist port; C, camera port; N, Nathanson liver hook; R, robotic arm; SPDP, spleen-preserving distal pancreatectomy

Robo-SPDP was performed using the hinotori system, following the Kimura technique for splenic vessel preservation. We placed the patient in the lithotomy position with a 13° head-up tilt and a 5° left-side-up tilt. We inserted 5 trocars, including one for the patient-side surgeon. Owing to the patient’s relatively small body habitus, the ports were positioned slightly caudally to maintain an optimal working distance from the target anatomy. The R3 port was used for the camera, whereas R1 and R2 served as the console surgeon’s left-hand ports (**Fig. 2A**).

Due to the hinotori system currently lacking integrated robotic vessel sealing devices, a hybrid approach was necessary: the division of the greater omentum from the transverse colon and the opening of the greater and lesser omental sacs were performed laparoscopically using a vessel sealing system. The operation unit was then docked from the patient’s left side. The arm base was rotated to align parallel to the line connecting the R1 and R4 ports, with an 8° upward and 5° left-upward tilt angle. The main robotic instruments included: bipolar Maryland forceps (Arm 4), bipolar fenestrated forceps (Arm 2), and Croce grasper forceps (Arm 1) (Medicaroid, Hyogo, Japan).

The procedure began with a superior approach to the pancreas. The SPA was identified and encircled with a vessel loop at its most accessible point. Dissection was then carried out along the SPA toward the splenic hilum. During this step, the SPV was also identified and exposed, allowing visualization of the pancreatic tail margin. Next, an inferior approach was performed. The serosa along the inferior border of the pancreas was widely dissected to reach the splenic hilum. Gerota’s fascia was preserved, and the pancreatic body and tail were mobilized. Once the SPV was visualized dorsally, dissection between the SPV and the pancreatic parenchyma was continued until it connected with the superior dissection plane. Once the pancreatic tail was completely separated from the splenic hilum, it was rotated clockwise to facilitate dissection of the remaining attachments between the pancreatic parenchyma and the SPA/SPV. Based on the diagnosis of MCN, we considered that dissection around the superior mesenteric vein, portal vein, or common hepatic artery—which is typically performed for pancreatic body or tail cancer—was unnecessary. Achieving an R0 resection would be enough for this patient. Therefore, the pancreatic transection line was determined approximately 10 mm proximal to the tumor edge using intraoperative ultrasonography. After confirming a safe resection margin, parenchymal transection was performed using a linear stapler (Tri Staple 2.0 Black Intelligent Reload, 60 mm; SIG60AXT; Medtronic plc, Dublin, Ireland).

For this step, as the R4 port had the appropriate direction for inserting the stapler, it was converted to a 15-mm trocar, and Arm 4 was temporarily removed to allow insertion of the stapler by a patient-side surgeon (**Fig. 2B**). The resected specimen was placed in a plastic retrieval bag and extracted through the umbilical port, which was enlarged to 3 cm. Finally, the Robo-SPDP following the Kimura technique was completed (**Fig. 2C**). The total operative time was 376 min, with a console time of 319 min. Estimated blood loss was 30 mL.

**Fig. 3:**
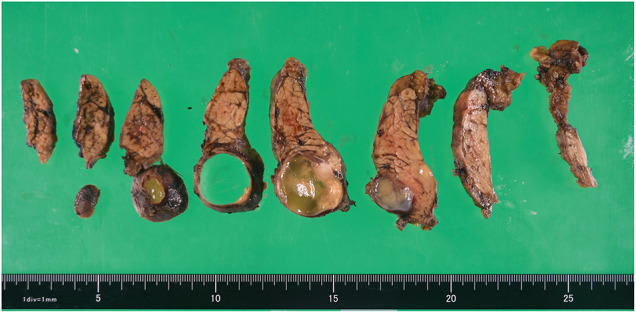
Gross appearance of the resected specimen.

Macroscopically, the lesion exhibited a cyst-in-cyst configuration, without solid components. The cyst contained serous fluid, and the inner surface was smooth, without mural nodules or papillary projections (**Fig. 3**).

**Fig. 4:**
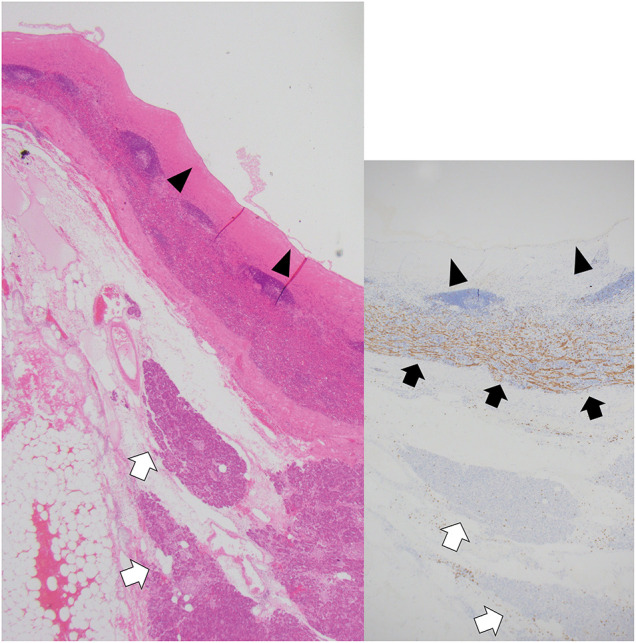
Histological findings (×40). The HE stains (left panel) and CD8 immunostaining (right panel) show a cyst lined by flattened to columnar epithelium (black arrowheads) overlying hyalinized stroma and splenic sinus structures. CD8-positive splenic tissue is observed in the surrounding area (black arrows), supporting the diagnosis of an epithelial cyst arising from an IPAS. The white arrows indicate pancreatic tissue. HE, hematoxylin and esoin; IPAS, intrapancreatic accessory spleens

Histologically, the cyst was lined by a single layer of flattened to columnar epithelium, underlain by a hyalinized fibrous stroma. Notably, the ovarian-type stroma characteristic of MCN was absent. Immunohistochemical analysis revealed a CD8-positive splenic sinus structure, confirming the diagnosis of an ECIPAS (**Fig. 4**).

Her postoperative course was uneventful, and she was discharged on POD 10. Follow-up imaging at 3 months showed no recurrence with preserved SPA and SPV perfusion.

## DISCUSSION

We report a rare case of ECIPAS that had been preoperatively misdiagnosed as MCN. Histologically, ovarian-type stroma—a characteristic feature of MCN—was absent, whereas CD8 immunostaining confirmed the presence of splenic tissue, leading to a definitive diagnosis of ECIPAS. In this case, we successfully performed minimally invasive Robo-SPDP using hinotori Surgical Robot System.

ECIPAS is an uncommon entity, first described by Davidson et al in 1980.^5)^ A PubMed search identified only 43 published articles to date, the majority of which were individual case reports. Despite recognition of this condition, standardized diagnostic criteria have not been established, and preoperative diagnosis remains challenging.

Differentiating ECIPAS from MCNs and other pancreatic cystic neoplasms is critical. A key imaging clue is the presence of solid components adjacent to the cyst that enhance similarly to splenic tissue on dynamic CT or MRI, indicative of an accessory spleen.^6,7)^ SPIO-enhanced MRI can aid in diagnosis, as SPIO is taken up by reticuloendothelial cells in the liver and spleen, including accessory spleens, resulting in signal loss on T2-weighted images.^8)^ Similarly, technetium-99m sulfur colloid scintigraphy can demonstrate radiotracer uptake in the splenic tissue, although its use is limited in routine practice.^2)^ Endoscopic US (EUS) also provides high-resolution visualization of cyst wall architecture, surpassing CT and MRI in structural detail.^9)^ When contrast-enhanced EUS demonstrates vascular patterns within the cyst wall that resemble splenic tissue, it may facilitate differentiation of ECIPAS from other cystic lesions. Additionally, when feasible, EUS-guided fine-needle aspiration allows cytological confirmation.^10)^ In our case, however, the accessory splenic tissue was markedly attenuated due to cystic expansion, obscuring its characteristic imaging features and further complicating preoperative differentiation from pancreatic neoplasms. A “cyst-in-cyst” appearance—typically considered characteristic of MCN—was observed and initially led to misinterpretation. Nevertheless, it is important to recognize that ECIPAS lesions can occasionally exhibit similar features.^11)^

Furthermore, the lesion increased in size by 12 mm over 2 years. Although epithelial cysts may gradually enlarge due to fluid accumulation, quantitative data on their growth kinetics remain limited. In contrast, for intraductal papillary mucinous neoplasms, a size increase of ≥5 mm over 2 years is considered a “worrisome feature” suggestive of malignant potential.^12)^ From this perspective, malignancy cannot be excluded in our case.

Historically, most ECIPAS cases were treated with open distal pancreatectomy and splenectomy, likely due to the limited availability of minimally invasive techniques. However, with advances in technology, the proportion of SPDP has increased, shifting from open^13,14)^ to laparoscopic approaches.^15–22)^ To date, including our case, only 3 reports have described Robo-SPDP for ECIPAS.^23,24)^ Although SPDP is technically demanding, preserving the spleen offers immunological benefits and reduces the risk of infectious complications.^25,26)^ Moreover, regarding the pancreatic parenchymal resection line, because this patient was diagnosed with an MCN, a noninvasive low-grade malignancy, preservation of residual pancreatic function was also an important consideration. Previous studies have shown that resection of more than 25% of pancreatic volume is associated with a higher risk of postoperative endocrine dysfunction.^27)^ Therefore, an R0 resection margin was considered sufficient, allowing us to maximize preservation of the pancreatic remnant in this patient.

Given the unavailability of the da Vinci surgical system at our institution, we refer to Uchida et al.’s detailed comparison of hinotori and da Vinci distal pancreatectomy techniques.^28)^ Due to the lack of integrated energy devices in the hinotori system, we adopted a “2-surgeon” technique, in which the patient-side surgeon performed vessel sealing or stapling using laparoscopic instruments.^29,30)^ Notably, the hinotori system features a docking-free design; therefore, when an arm is removed—such as during pancreatic parenchymal transection—the corresponding port becomes immediately available for use by the patient-side surgeon. In this case, both the R2 and R4 ports were considered preoperatively for stapler insertion, and the R4 port provided the most suitable trajectory for safe stapler delivery based on the patient’s body habitus and the planned pancreatic transection line. Owing to the docking-free design of the hinotori system, this flexibility in port selection will underscore its versatility for a patient-side surgeon. This maneuver may be difficult with the da Vinci system, as its ports remain docked to the robotic arm.

Two main techniques exist for SPDP: the Kimura technique, which preserves both the SPA and SPV, and the Warshaw technique, which sacrifices these vessels and relies on collateral circulation. Although the Kimura technique is technically more demanding and often associated with longer operative times, it carries a lower risk of postoperative splenic infarction, abscess formation, and perigastric varices.^31–33)^

A limitation of this report is its nature as a single case study, which inherently limits the generalizability of findings regarding the efficacy and safety of robotic surgery or the diagnostic approach. A recent single-center retrospective study comparing laparoscopic and robotic SPDP reported that robotic SPDP was associated with significantly reduced intraoperative blood loss, lower conversion rates, and higher success rates of splenic vessel preservation.^34)^ Further studies, including larger series or prospective trials, are needed to validate these observations.

## CONCLUSIONS

This case highlights the importance of considering ECIPAS in the differential diagnosis of pancreatic cystic lesions, particularly when imaging findings mimic cystic neoplasms. Surgical resection is recommended, and minimally invasive spleen-preserving distal pancreatectomy is an appropriate treatment. Furthermore, our experience suggests that robotic assistance, using the hinotori system in this case, appears to be a safe and effective surgical approach facilitating precise dissection and reliable preservation of the splenic vessels.

## DECLARATIONS

### Availability of data and materials

The datasets supporting the conclusions of this article are included within the article.

## References

[1] Halpert B, Gyorkey F. Lesions observed in accessory spleens of 311 patients. Am J Clin Pathol 1959; 32: 165–8. 13670140 10.1093/ajcp/32.2.165

[2] Spencer LA, Spizarny DL, Williams TR. Imaging features of intrapancreatic accessory spleen. Br J Radiol 2010; 83: 668–73. 19690077 10.1259/bjr/20308976 PMC3473517

[3] Läuffer JM, Baer HU, Maurer CA, et al. Intrapancreatic accessory spleen. A rare cause of a pancreatic mass. Int J Gastrointest Cancer 1999; 25: 65–8. 10.1385/IJGC:25:1:65 10211424

[4] Li BQ, Lu J, Seery S, et al. Epidermoid cyst in intrapancreatic accessory spleen: A systematic review. Pancreatology 2019; 19: 10–6. 30366677 10.1016/j.pan.2018.10.008

[5] Davidson ED, Campbell WG, Hersh T. Epidermoid splenic cyst occurring in an intrapancreatic accessory spleen. Dig Dis Sci 1980; 25: 964–7. 7449592 10.1007/BF01308048

[6] Ding Q, Ren Z, Wang J, et al. Intrapancreatic accessory spleen: evaluation with CT and MRI. Exp Ther Med 2018; 16: 3623–31. 30250526 10.3892/etm.2018.6613 PMC6144032

[7] Kawamoto S, Johnson PT, Hall H, et al. Intrapancreatic accessory spleen: CT appearance and differential diagnosis. Abdom Radiol 2012; 37: 812–27. 10.1007/s00261-011-9830-x 22160284

[8] Kim SH, Lee JM, Han JK, et al. MDCT and superparamagnetic iron oxide (SPIO)-enhanced MR findings of intrapancreatic accessory spleen in seven patients. Eur Radiol 2006; 16: 1887–97. 16547707 10.1007/s00330-006-0193-6

[9] Tsujimoto R, Kurokawa R, Yamamoto A, et al. Epidermoid cyst in an intrapancreatic accessory spleen complicating clinical decision-making: a case report with characteristic imaging findings. Cureus 2024; 16: e69957. 39445281 10.7759/cureus.69957 PMC11496593

[10] Minami R, Nakahodo J, Tabata H, et al. Epidermoid cyst in an intrapancreatic accessory spleen diagnosed by contrast-enhanced EUS and EUS-FNA (with video). Endosc Ultrasound 2024; 13: 49–51. 38947112 10.1097/eus.0000000000000047 PMC11213605

[11] Hirata Y, Arisaka Y, Kutsumi H, et al. A case of epidermoid cyst in an intrapancreatic accessory spleen showing cyst-in-cyst-like structure mimicking mucinous cystic neoplasm. Nihon Shokakibyo Gakkai Zasshi 2015; 112: 1858–67. (in Japanese) 26440689 10.11405/nisshoshi.112.1858

[12] Tanaka M, Fernandez-del Castillo C, Adsay V, et al. International consensus guidelines 2012 for the management of IPMN and MCN of the pancreas. Pancreatology 2012; 12: 183–97. 22687371 10.1016/j.pan.2012.04.004

[13] Kanazawa H, Kamiya J, Nagino M, et al. Epidermoid cyst in an intrapancreatic accessory spleen: a case report. J Hepatobiliary Pancreat Surg 2004; 11: 61–3. 15754048 10.1007/s00534-003-0844-9

[14] Kadota K, Kushida Y, Miyai Y, et al. Epidermoid cyst in an intrapancreatic accessory spleen: three case reports and review of the literatures. Pathol Oncol Res 2010; 16: 435–42. 19949910 10.1007/s12253-009-9229-y

[15] Itano O, Chiba N, Wada T, et al. Laparoscopic resection of an epidermoid cyst originating from an intrapancreatic accessory spleen: report of a case. Surg Today 2010; 40: 72–5. 20037845 10.1007/s00595-009-4006-9

[16] Iwasaki Y, Tagaya N, Nakagawa A, et al. Laparoscopic resection of epidermoid cyst arising from an intrapancreatic accessory spleen: a case report with a review of the literature. Surg Laparosc Endosc Percutan Tech 2011; 21: e275–9. 22002295 10.1097/SLE.0b013e31822dd14a

[17] Urakami A, Yoshida K, Hirabayashi Y, et al. Laparoscopy-assisted spleen-preserving pancreatic resection for epidermoid cyst in an intrapancreatic accessory spleen. Asian J Endosc Surg 2011; 4: 185–8. 22776306 10.1111/j.1758-5910.2011.00102.x

[18] Khashab MA, Canto MI, Singh VK, et al. Endosonographic and elastographic features of a rare epidermoid cyst of an intrapancreatic accessory spleen. Endoscopy 2011; 43 Suppl 2 UCTN: E193–4. 21590599 10.1055/s-0030-1256272

[19] Panagiotopoulos N, Acharya M, Ahmad R, et al. Epithelial inclusion cyst arising within an intra-pancreatic splenunculus. Int J Surg Case Rep 2012; 3: 118–20. 22288063 10.1016/j.ijscr.2011.12.005 PMC3267291

[20] Zhou B, Zhang Q, Zhan C, et al. Laparoscopic spleen-preserving pancreatic resection for epidermoid cyst in an intrapancreatic accessory spleen: case report and literature review. Ther Clin Risk Manag 2018; 14: 937–44. 29805263 10.2147/TCRM.S165489 PMC5960247

[21] Lo CH, Tsang PM, Cheng SY, et al. Epidermoid cyst in an intrapancreatic accessory spleen with abnormally high CEA level in cyst fluid: a case report. Autops Case Rep 2022; 12: e2021369. 35496737 10.4322/acr.2021.369 PMC9037849

[22] Bhutiani N, Egger ME, Doughtie CA, et al. Intrapancreatic accessory spleen (IPAS): a single-institution experience and review of the literature. Am J Surg 2017; 213: 816–20. 27894508 10.1016/j.amjsurg.2016.11.030

[23] van Dijck WP, Groot VP, Brosens LA, et al. Rare case of an epithelial cyst in an intrapancreatic accessory spleen treated by robot-assisted spleen preserving distal pancreatectomy. Case Rep Gastrointest Med 2016; 2016: 9475897. 27847657 10.1155/2016/9475897 PMC5099494

[24] Tan HJ, Neo WL, Lee SY, et al. Epidermal inclusion cyst in an intra-pancreatic accessory spleen: a differential diagnosis for pancreatic cystic neoplasms and review of the literature. J Gastrointest Cancer 2019; 50: 308–14. 28889365 10.1007/s12029-017-0002-2

[25] Shi N, Liu SL, Li YT, et al. Splenic preservation versus splenectomy during distal pancreatectomy: a systematic review and meta-analysis. Ann Surg Oncol 2016; 23: 365–74. 26493758 10.1245/s10434-015-4870-z

[26] Nakata K, Shikata S, Ohtsuka T, et al. Minimally invasive preservation versus splenectomy during distal pancreatectomy: a systematic review and meta-analysis. J Hepatobiliary Pancreat Sci 2018; 25: 476–88. 29943909 10.1002/jhbp.569

[27] Kang JS, Jang JY, Kang MJ, et al. Endocrine function impairment after distal pancreatectomy: incidence and related factors. World J Surg 2016; 40: 440–6. 26330237 10.1007/s00268-015-3228-9

[28] Uchida Y, Takahara T, Kawase T, et al. Robotic distal pancreatectomy using the hinotori surgical system: differences in surgical techniques from the daVinci surgical system. Surg Oncol 2025; 59: 102195. 39970600 10.1016/j.suronc.2025.102195

[29] Takagi K, Fuji T, Yasui K, et al. Robotic distal pancreatectomy using two-surgeon technique (TAKUMI-4): a technical note and initial outcomes. Langenbecks Arch Surg 2025; 410: 171. 40455094 10.1007/s00423-025-03751-3 PMC12130140

[30] Kakiuchi Y, Kuroda S, Yoshida Y, et al. An effective surgical educational system in the era of robotic surgery: “Double-Surgeon Technique” in robotic gastrectomy for minimally invasive surgery. Langenbecks Arch Surg 2024; 410: 20. 39731598 10.1007/s00423-024-03593-5 PMC11682005

[31] Maehira H, Tani M, Mori H, et al. Long-term outcomes after spleen-preserving distal pancreatectomy with splenic vessels preservation or resection: a nationwide survey of the Japanese Society of Pancreatic Surgery. Surgery 2024; 175: 1570–9. 38519409 10.1016/j.surg.2024.01.027

[32] Granieri S, Bonomi A, Frassini S, et al. Kimura’s vs Warshaw’s technique for spleen preserving distal pancreatectomy: a systematic review and meta-analysis of high-quality studies. HPB (Oxford) 2023; 25: 614–24. 36941150 10.1016/j.hpb.2023.02.009

[33] Watanabe Y, Nakata K, Abe T, et al. Short- and long-term outcomes after minimally invasive spleen-preserving distal pancreatectomy with and without preservation of the splenic vessels: splenic vessel-preserving procedure is a “double-edged sword” in left-sided portal hypertension. Surg Endosc 2025; 39: 8197–208. 40960541 10.1007/s00464-025-12167-5

[34] Ami K, Kamei K, Nakano M, et al. A comparison of laparoscopic and robotic distal pancreatectomy with spleen and Splenic vessels preservation: an intention-based evaluation in a single-center retrospective study. Surg Today 2026; 56: 799–806. 41263982 10.1007/s00595-025-03190-z

